# Curriculum Innovations: Enhancing Skills in Serious Illness Communication in Neurology Residents Using Simulation: A Pilot Study

**DOI:** 10.1212/NE9.0000000000200140

**Published:** 2024-08-06

**Authors:** Marcey Osgood, Brian Silver, Jennifer Reidy, Vandana Nagpal

**Affiliations:** From the Department of Neurology (M.O.), Lahey Health and Medical Center, Burlington, MA; and Department of Neurology (B.S.), and Department of Palliative Care (J.R.,V.N.), University of Massachusetts Chan Medical School, Worcester.

## Abstract

**Background and Problem Statement:**

Patients with acute ischemic stroke are faced with prognostic uncertainty, progressive decline, and early mortality. Many neurologists report a lack of education and experience in providing palliative care. We developed a simulation-based curriculum to improve residents' confidence and comfort with conducting late-stage goals of care (GOC) conversations.

**Objectives:**

To assess and improve neurology residents' self-reported confidence and comfort around GOC discussions, prognostication, and hospice; encourage neurology residents to conduct GOC conversations early in the illness; introduce neurology residents to a structured framework for conducting GOC conversations; facilitate the residents to build rapport and convey a mindful presence during GOC conversations; provide direct, real-time feedback and an opportunity for redo and practice; and identify gaps for education.

**Methods and Curriculum Description:**

The 3-hour experience included a didactic session followed by an interactive simulation and debriefing. The residents' objectives were to deliver difficult news, discuss prognosis, explore goals, navigate treatment options, and discuss end-of-life care including hospice. The faculty observed each interaction and called time-outs to allow the residents to self-assess and obtain feedback. Residents and faculty debriefed to identify take-home points and to reflect on their emotions, self-care, and sense of purpose in medicine.

**Results and Assessment:**

Twenty-six neurology residents filled out an anonymous presurvey to self-assess their confidence and comfort surrounding GOC conversations. More than 50% of residents reported being confident in conducting GOC discussions, whereas only 42% reported adequate prior training. Postsession, more than 90% of residents reported that training was relevant, helpful, organized, and clear. Faculty identified that residents had difficulty addressing prognosis, assessing goals, planning treatment, and using silence, responding to emotion, and displaying empathy. Fifteen residents filled out a postsurvey that revealed improved comfort with delivering prognosis, discussing hospice, and initiating early GOC discussions.

**Discussion and Lessons Learned:**

Our project uniquely focuses on late-stage GOC conversations and builds on existing literature that supports a structured program with both didactic and simulation components to improve residents' abilities to effectively navigate GOC conversations with patients and families. Future work will focus on reinforcement and reassessment of communication skills.

## Introduction and Problem Statement

Neurologic diseases are largely incurable, lead to decreased life expectancy, and cause a multitude of signs and symptoms that negatively affect quality of life. Caregivers for those with neurologic diseases also have high rates of distress and burnout.^1^ This is particularly apparent for families of patients with acute ischemic stroke. Stroke remains a leading cause of both mortality and disability in the United States with greater than 50% of stroke-related deaths occurring in the hospital.^2^ For patients admitted with stroke, the neurologist is uniquely positioned to provide primary palliative care (PPC) and facilitate goals of care (GOC) conversations from the time of diagnosis.^3,4^ The American Heart and Stroke Association recognizes the enormous palliative care (PC) needs of stroke patients and recommends that all patients adversely affected by stroke have access to PPC early in the disease course.^2^ Furthermore, PC education should be a priority for neurology programs.^2,3^ However, research continues to show a gap in education on topics of PC and many neurologists report feeling ill equipped to deliver PPC to their patients.^5-7^

Palliative medicine is specialized medical care for patients with serious illness that focuses on symptom relief, expert communication, emotional support, and care coordination to improve quality of life for both patients and caregivers.^1^ Although the Accreditation Council for Graduate Medical Education (ACGME) requires that residency training programs provide education in PC, a 2009 survey revealed 52% of neurology programs offered a didactic with 7.8% offering a PC clinical rotation.^8,9^ More recent data from 2018 showed persistent gaps, with more than half of neurology residents reporting insufficient PC education and 1 in 5 residents reporting none.^10^ Residents also report variable exposure to GOC conversations and a lack of training in prognostication, communicating with surrogate decision makers, and navigating multiple decision points during these complex conversations.^11,12^ Research also shows that programs often provide PC lectures and seminars without hands-on practice or other engaged learning approaches.^10,13^ Furthermore, throughout the course of clinical training, residents receive limited observation or direct feedback regarding GOC conversations.^11^

It remains unclear how to best provide communication skills education to neurology residents. Nationally, there are a number of evidence-based programs that use a structured framework with simulated patient scenarios and targeted feedback to improve learners' skills.^14-16^ Although these programs have shown success, they are not targeted to neurology patients who have unique needs. There is increasing interest in developing a curriculum for neurology trainees, with a growing body of literature.^17-22^ In review of previously published literature, the combination of time spent in didactic learning along with either role-play or standardized-patient exercises seemed to improve resident education and ability to conduct GOC conversations.^17-19^ In these prior studies, there were often several lengthy didactic sessions with limited time spent in intentional practice. We aimed to create an experience that was easily deliverable and replicable, in addition to retaining its educational value. Our residents had limited PC didactics and no formal training in GOC conversations based on evaluation of our program curriculum; hence, we incorporated elements from Kirkpatrick model of learning^23^ to create an educational program where residents could practice GOC conversations in a safe and constructive environment. The session had to fit within the time constraints of the residents, and session time was devoted so as to maximize deliberate practice. In conducting a needs assessment for our program, we noted that the residents spent most of their time in care of acutely ill neurologic patients admitted to the hospital. Our program has a large stroke service, and often, residents are expected to conduct and lead GOC conversations, sometimes in the absence of an attending. We developed this unique late-stage GOC conversation curriculum focused on practicing difficult conversations in acute ischemic stroke to meet this resident need and curricular gap. We endeavored to maximize allotted time (half-day) by creating 2-person groups, thereby allowing the experience of working with a team and learning through peer observation; we integrated increased practice time with actors with real-time feedback and debriefing. We designed the project to address levels 1–3 in Kirkpatrick model of learning.^23^

## Objectives

We developed GOC communication curriculum with an overarching goal to enhance neurology residents' confidence, comfort, and ability to conduct late-stage serious illness conversations effectively and compassionately early in the hospital course for a patient with acute ischemic stroke.

Program objectives:

1. Assess and improve neurology residents' self-reported confidence and comfort around GOC discussions, prognostication, and hospice.
2. Encourage neurology residents to conduct GOC conversations early in the course of illness.
3. Introduce neurology residents to a structured framework for conducting GOC conversations.
4. Facilitate the residents to build rapport and convey a mindful presence during GOC conversations through openness, deep active listening, pauses, and honesty.
5. Provide direct, real-time feedback and an opportunity for redo and practice.
6. Identify areas of need for further education.

## Methods and Curriculum Description

We designed and implemented a 3-hour communication skills training using the Serious Illness Conversation Guide (SICG) from Ariadne Labs.^15^ The focus of this simulation was to train neurology residents of all postgraduate levels, in describing prognosis, breaking difficult news, sitting with strong emotion, matching priorities with treatment preferences, and recommending hospice care for a patient with an ischemic stroke. We adapted best practices in communication from the Serious Illness Care Program and Vital Talk in addition to developing an evaluation tool using the American Academy of Hospice and Palliative Medicine SECURE framework.^14,15,24^

Briefly, the sessions were interactive learning experiences for neurology residents in the objective structured clinical examination format at our institution's simulation center. At the beginning of the session, there was a 60-minute introduction in which residents reflected on the value of having these challenging and emotional conversations. Trained faculty from neurology and PC led this interactive didactic session and reviewed prognostication in stroke patients, SICG with patient tested language, and a primer on hospice including language for introducing the concept to patients and families.

Next, residents were divided into groups of 2 and introduced to 2 actors portraying family members of a patient admitted with an ischemic stroke. Residents had 45 minutes in which they practiced various components of the GOC discussion using the SICG. Each group had a faculty facilitator observing the conversation. The faculty facilitator, residents, and actors participated in real-time feedback during the encounter and residents had the chance to focus on the specific skills they wanted to practice. After the simulation, all participants met with faculty for 60 minutes to debrief the experience and identify goals and strategies for deliberate practice in real-world patient care.

### Details of the Curriculum

#### Before Simulation

We identified and assembled a skilled group of neurology and PC physicians (faculty facilitators) who had an interest and experience in difficult conversations and simulation teaching. Three main faculty with expertise in PC led the design and implementation of this training. Two of the lead faculty were PC physicians with extensive expertise in GOC discussions and simulation. They were also Vital Talk trained and had conducted a similar project with the internal medicine residents at our institution.^25^ The other lead faculty member was a neurocritical care physician with extensive training in PC and simulation, having taken part in multiple prior workshops including Vital Talk. Other faculty joining the project (2 neurologists) had to go through a faculty development session and cofacilitation of a small group with one of the lead faculty until they were deemed to have an expertise in facilitating a group on their own. During cofacilitated sessions, each faculty evaluated the residents, and the observer faculty evaluations were compared with the lead faculty evaluations to review inter-rater differences. Given the extensive involvement of the lead faculty, the 2 other faculty never completed a session alone.

We also worked with our simulation center to schedule a group of experienced actors for this training. The materials including an introductory didactic, a faculty guide, patient case with character background, evaluation tools, and surveys were created via collaboration of faculty facilitators. The surveys, in fact, were like the ones used in previous study done by the faculty.^25^ The scheduling for simulation sessions was coordinated by the residency administrative assistant, neurology chief residents, faculty, and simulation center staff.

A few weeks before the first resident session, faculty facilitators and actors participated in a 2-hour faculty development meeting to orient everyone to the goals, objectives, teaching methods, and evaluation tools to standardize our teaching. During this meeting, the group reviewed the clinical case, evaluation materials, and roles. Specifically, the faculty facilitators were to guide the in-room conversations, call time-outs, and provide appropriate and timely feedback, whereas the actors portrayed the case with appropriate challenges and emotional reactions to prompt learning opportunities. The detailed patient case, character background, faculty guide, and evaluation tools are shown in eAppendix 1.

One week before the session, the residency program administrator sent a welcome email to residents to remind them of the training, including the session's goals and agenda. The email included a presurvey to collect baseline data on residents' self-assessed comfort and confidence in and frequency of GOC conversations (eAppendix 2). This presurvey was designed to help evaluate our first and second project objectives and also to facilitate our ability to assess Kirkpatrick levels 2 and 3.^23^ The survey also addressed the barriers residents perceived in conducting GOC discussions.

#### Day of Simulation

**Figure 1:**
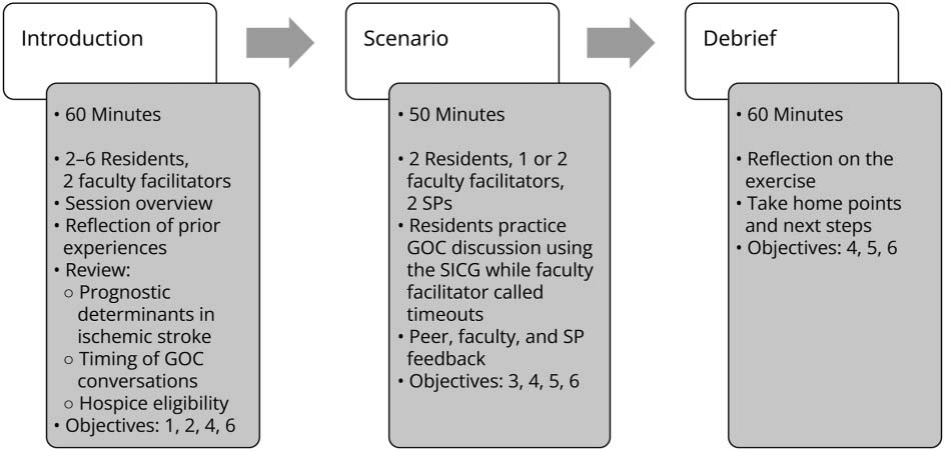
Curriculum Overview GOC = goals of care; SICG = Serious Illness Conversation Guide; SP = senior physician.

The total length of the session was 3 hours (Figure 1). The training started with 60-minute introduction. This took place in a large conference room with 2 faculty facilitators and 4 residents. The residents received a session overview and reflected on their prior experiences and barriers to communication. An interactive presentation reviewed the case, prognostication in acute ischemic stroke, timing of GOC discussions, and hospice eligibility. The residents were also introduced to SICG and practiced the patient-tested language. This introduction session allowed us to address objectives 1, 2, and 3. Please see eAppendix 3 for details on the introduction session (eAppendix 3).

Following the introduction, the residents entered the simulated scenario which took place over 50 minutes. There was a brief huddle to review the case and plan the encounter (eAppendix 4). Two residents with 1–2 faculty facilitators entered the exam room where 2 actors representing patient's family members were sitting. One of the residents began the interview using the SICG. The faculty facilitator called time-outs at points of need or around 5–6 minutes and asked for the resident's impressions. In addition to resident self-assessment, the facilitator also solicited feedback from the peer resident and actors. The facilitator then offered the first resident a chance to re-enter the scenario to try a different approach or to continue to encounter. Halfway through the session and/or after prognosis sharing step of SICG, the second resident continued the interview, and the faculty facilitator repeated the time-out and feedback process as described. With the remaining time, the residents were allowed to practice any part of the SICG they felt they needed more practice on. This simulated scenario was designed to evaluate objectives 1, 3, 4, and 5.

Following the scenario, the residents and faculty facilitators met in the conference room to debrief. This 60-minute debrief was a time to reflect on the exercise, discuss self-care, and review take-home points, along with next steps. This was followed by 10 minutes in which the residents and faculty filled out an evaluation.

#### Evaluation

As above, the faculty and actors provided concrete, real-time feedback to the residents during the simulated encounter. Faculty then evaluated the performance of each resident based on the content of the GOC discussion as well as the interpersonal skills of the resident (eAppendix 1). The faculty noted whether a task was “done,” “partially done,” or “not done.” “Partially done” was when the faculty felt the task was addressed inadequately but was attempted. The residents evaluated the effectiveness of the session at the end of the training and provided written feedback to guide programmatic and pedagogical improvements. The residents were asked to rate the study materials, introduction session, simulation scenario, and the debrief session along with listing 3 takeaway points (eAppendix 1). These program evaluations allowed us to further address objectives 3, 4, 5, and 6. The evaluations also helped us to evaluate Kirkpatrick level 1.^23^

#### After Simulation

The residency administrative assistant sent emails to residents at 1, 3, 6, and 12 months after the training with brief postsurveys to evaluate the impact on their everyday practice over time. The emails also had “refreshers” or pearls for deliberate practice using content from the Serious Illness Care Program and Vital Talk (eAppendix 5). The postsurvey allowed us to further assess objectives 1 and 2 along with the evaluation of Kirkpatrick levels 2 and 3.^23^

### Standard Protocol Approvals, Registration, and Patient Consents

This study was reviewed by the Institutional Review Board (IRB) at the UMass Chan Medical School and was determined not to be human research. The need for consent was waived (IRB number: H00015188).

### Data Availability

Data will be made available on request directed to the corresponding author.

### Program Cost Estimates

The faculty volunteered their time and were not paid to participate in this curriculum. The cost attributed to holding this program is related to the cost of reserving simulation center rooms, SPs, and support staff. For our simulation center, 1 session with 2 residents, 2 actors, and 1 simulation room was approximately 600 US dollars. We were able to train an entire residency class (7 residents) for approximately 2000 US dollars which reflects the yearly cost attributed to this training.

## Results and Assessment Data

### Learner Characteristics

We completed 10 simulation sessions with a total of 30 neurology residents from 2018 to 2020. At our institution, there are 7 residents in each postgraduate year (PGY) (2, 3, and 4). Participating in this study were 13 second-year residents, 8 third-year residents, and 9 fourth-year residents. Twenty-seven residents completed the presurvey although 1 respondent did not answer any of the survey questions except for their role, age, and sex and was not included in the final survey results (N is 26 with a response rate of 86.6%). The learner characteristics of those responding to the survey are shown in Table 1. Of note, the survey asked the residents to report their sex as male or female which is reported in Table 1. All 26 residents reported sex although only 24 respondents reported the PGY level. Table 1 shows an N of 24 to reflect those reporting the PGY level although for reported sex, N is 26. Fifty percent of the presurvey respondents were PGY 2, 16% PGY 3, and 33.3% PGY 4. The average age of the presurvey respondents was 31 years and 46% were female. Fifteen residents submitted the postsurvey (response rate 50%). Of these 15, 1 did not report their PGY level, age, or sex; so, for the learner characteristics in Table 1, N is 14. It should be noted that the postsurveys were filled out at least 1 month following the simulation, at which time some residents had graduated to the next PGY level. This is reflected in the fact that in the postsurvey, only 14% of the respondents were PGY 2 level, whereas 35% are PGY 3 and 50% are PGY 4. Half postsurvey respondents were female with an average age of 32 years (Table 1).

**Table 1:** Respondent Characteristics at the Time of Survey

Respondent characteristics	Presimulation survey (n = 24), n (%)	Postsimulation survey (n = 14), n (%)
Postgraduate year 2	12 (50)	2 (14.2)
Postgraduate year 3	4 (16.6)	5 (35.7)
Postgraduate year 4	8 (33.3)	7 (50)
Average age, y	31	32
Sex, female	12/26 (46.1)	7 (50)

Twenty-six residents responded to the survey, although only 24 reported the postgraduate year. N for the postgraduate year is 24, although N for the sex is 26. The respondents were asked to self-identify sex as either male or female.

### Presurvey

**Figure 2:**
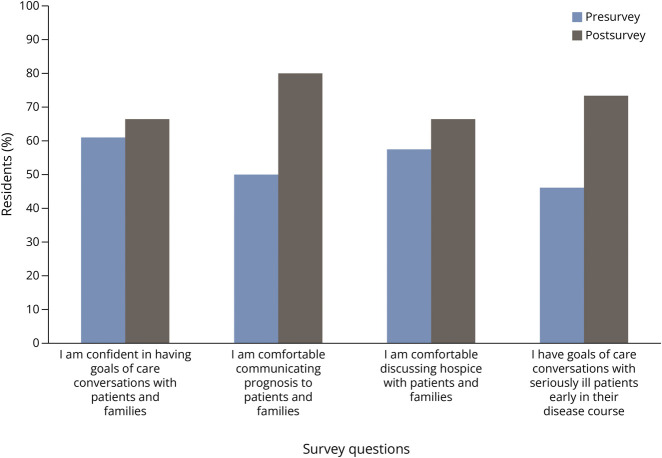
Presurvey and Postsurvey Results The y axis is the percentage of residents that agree and strongly agree with the statements noted on the survey. The x axis represents the specific survey question. The total number of residents responding to the presurvey is 26 and the postsurvey is 15.

The presurvey was designed to evaluate program objective 1 and assess the residents' confidence and comfort in conducting GOC discussions. The presurvey was anonymous and 26 residents filled out the presurvey fully. Learner responses to the presurvey are summarized in Figure 2. Sixty-one percent of the residents reported having confidence with conducting GOC discussions, 57.6% were comfortable discussing hospice care, and 42% felt adequately prepared by their prior training. The comfort with discussing prognosis was 50%. The survey also revealed that 53.9% of residents did not engage in GOC discussions early in the disease course. Sixty-five percent of residents reported having 1 or fewer GOC conversations in a week. When asked to identify personal barriers that make GOC conversations difficult, the most reported themes included unclear/uncertain prognosis, cultural awareness/differences, and concern around emotional toll on the families.

### Program Evaluation

Immediately following the simulation, 29 of 30 residents completed a program evaluation in which they rated the simulation experience. Table 2 summarizes the program evaluation. Over 90% of the residents reported being satisfied or very satisfied with the overall goals, relevancy, and delivery of the simulation. Some common themes emerged when reading the resident-reported takeaway points. Eleven residents (38%) commented on learning to use silence as a tool, including one who remarked “silence is as valuable as words.” Another theme was using clear, concise, and direct language which was noted by 17 residents (59%). Nine residents (31%) saw the value in the SICG, and many reported that having a structured format with patient-tested language was important. Six (21%) of the residents noted that showing empathy and responding to emotion was important to build a connection with the surrogate decision makers.

**Table 2:** Resident Evaluation of the Simulation

Questionnaire statements	Postsimulation program evaluation responses satisfied or very satisfied (%)
Study materials provided relevant information	100
Study materials were helpful	100
Goals of the introduction were clear	100
Introduction session provided relevant information	100
Introduction session was organized	96.5
Goals of the simulation scenario were clear	93.1
Standardized patient portrayal was realistic	100
Simulated experience was relevant	96.5
Goals of the debrief were clear	100
Debrief provided relevant feedback	100
Debrief was organized	100

### Faculty Evaluation

The results of this evaluation are reported in Table 3. Given the structure of the simulation with 2 residents taking part in the same GOC discussion, not all residents completed every task on the evaluation checklist. This is reflected in the table where the number of residents completing a particular task is not equal to 30. More than 70% of residents did not address or inadequately addressed patient values and priorities, prognosis and treatment options, and development of a plan with recommendations based on patient wishes. Despite the high level of confidence noted on the presurvey, residents struggled with these components of GOC conversation and with using medical jargon. Residents were made aware of their areas of deficiency during the time-outs and feedback throughout the session and were given the opportunity to practice. They were not provided a written evaluation or given a learning plan based on this evaluation. The debrief focused on the gaps noted by the faculty, and there was a discussion surrounding what the residents learned about their own abilities.

**Table 3:** Faculty Evaluation of the Residents Based on the Content and the Quality of the Communication

Content evaluation	Task fully completed, N (%)	Task partially completed, N (%)	Task not done, N (%)
Greets patient/family member and introduces self and team (N = 30)	28 (93.3)	2 (6.6)	0 (0)
Explains the purpose of the meeting (N = 26)	15 (57.6)	8 (30.7)	3 (11.5)
Asks the patient/family to describe their understanding of the patient's illness (N = 24)	16 (66.6)	6 (25)	2 (8.3)
Describes the current medical condition without jargon (N = 28)	10 (35.7)	16 (57.1)	2 (7.1)
Offers an opportunity for the patient/family to ask questions (N = 30)	12 (40)	14 (35)	4 (13.3)
Explores patient's values and priorities (N = 17)	3 (17.6)	14 (82.3)	0 (0)
Discusses prognosis and treatment options including hospice (N = 29)	7 (24.1)	17 (58.6)	5 (17.2)
Develops a plan of care based on priorities and makes a recommendation (N = 24)	6 (25)	11 (45.8)	7 (29.1)
Discusses next steps and follow-up (N = 19)	6 (31.5)	5 (26.3)	8 (42.1)

Communication evaluation (N = 30)	Excellent, N (%)	Satisfactory, N (%)	Unsatisfactory, N (%)
Maintains an open posture	21 (70)	10 (33.3)	0 (0)
Assures comfort and privacy	17 (56.6)	13 (43.3)	0 (0)
Assumes a comfortable interpersonal communication distance	17 (56.6)	13 (43.3)	0 (0)
Allows patient and family time to reflect, does not rush	8 (26.6)	9 (30)	13 (43.3)
Recognizes and responds to emotion	5 (16.6)	13 (43.3)	12 (40)
Displays empathy through words and expressions	8 (26.6)	16 (53.3)	6 (20)

Because the residents participated in groups, not every resident was able to be evaluated on each task. The number of residents evaluated on a particular task is denoted next to the task description.

Overall, the faculty determined that 13% (4 out of 30) of the residents were able to independently have GOC discussions. Three of the 4 residents deemed competent to have independent GOC conversations were PGY 4 residents. The remainder (87%) required further supervision and instruction to effectively conduct GOC discussions on their own. We were unable to match the residents to their presurvey responses given that the presurvey was anonymous. It remains unclear whether those that were deemed independent scored higher on their level of comfort or confidence on presurvey.

### Postsurvey

A total of 15 residents filled out the postsurvey with 9 residents at 1 month, 5 residents at 3 months, and 1 resident at 6 months. The postsurvey included the residents' email address, and the surveys returned at 1, 3, and 6 months were all from different residents. The results for the postsurvey were pooled and are shown in Figure 2. Confidence in conducting GOC conversations increased from 61.5% (16/26) on the presurvey to 66.6% (10/15) on the postsurvey. The comfort with discussing prognosis increased from 50% (13/26) on the presurvey to 80% (12/15) on the postsurvey. The number reporting early GOC discussions also increased from 46% (12/26) on the presurvey to 73% (11/15) on the postsurvey. All 15 of the residents reported that the simulation improved their ability to conduct GOC discussions.

## Discussion and Lessons Learned

This curriculum was developed to address the lack of PC training during neurology residency and meets the ACGME requirements and milestones for PC training^9^ along with multiple American Academy of Neurology–proposed competencies for a neuropalliative curriculum.^26^ Our curriculum uniquely combines multiple evidence-based teaching methods including a small group interactive didactic, skills practice in a safe and simulated environment, a standardized patient encounter, direct observation, peer reflection, and real-time faculty feedback.^27^ We chose to focus this curriculum on late-stage GOC conversations in acute stroke patients because our residents spend significant time in care of this patient population, which could help facilitate clinical practice following this curriculum. The use of an acute patient scenario also allowed us to review some of the challenges of neuropalliative care including the abrupt change in patient function, uncertainty of prognosis, and discussion with a surrogate decision maker when the patient is unable to participate. We were also able to introduce the concept of hospice care for patients with goals focused on comfort. This program addresses core skills of communicating prognosis with compassion and honesty, shared decision-making through assessing values and quality of life, and tailoring treatment to patients' unique goals and medical context.^28^ We used a single session and were able to keep the groups small to maximize the time spent in deliberate practice. We were also able to directly evaluate the residents' performance and provide real-time faculty and peer feedback.

It has been shown that time and experience alone don't produce improvement in physician communication, but specific skills training has an impact.^17-19,29-32^ Similar to prior studies, we found that a large number of residents reported confidence in having GOC conversations despite limited prior exposure or experience.^17-19^ Most residents were found to have substantial gaps in knowledge and skills and were deemed to require supervision and ongoing feedback. Dunning and Kruger described a similar phenomenon in which “incompetent” learners with minimal experience or exposure to a topic may overestimate their abilities.^33^ This overconfidence may be due to a lack of metacognition which also hinders the learner's insight into their true level of performance and their ability to self-reflect. David Davis further elucidated this concept and noted that self-assessments have limited accuracy, and resident knowledge and skills may be best evaluated through direct observation.^34,35^ This is an important learning point because programs often expect the residents to learn through apprenticeship and be competent after limited exposure. Residents may rely heavily on this method of training and be unaware of their limitations and gaps of knowledge. This impedes further learning because residents may fail to seek out supplementary education and may not respond to feedback. Our curriculum design may address this issue by incorporating a didactic component to increase the knowledge base along with direct observation, targeted, high-quality feedback, intentional practice, and debriefing which should not only increase the competency of the learner but also increase their ability for future self-assessment and receptivity to future feedback.^34,36-39^

Overall, this educational program was universally well-received by the residents; it was thought to be clear, organized, and relevant. Postsurvey results showed a slight increase in confidence conducting GOC discussions post-training. This may be due in part to residents overrating themselves initially and recognizing their knowledge gaps during the training.^33^ There was a larger increase in both comfort in discussing hospice and comfort in delivering prognosis on the postsurvey which were focuses of our curriculum. The increase in residents' reported comfort in delivering prognosis is likely from the discussion and practice on formulating prognosis, managing uncertainty, and prognosis communication practice during the simulation session.

Studies show that hospital-based referrals from neurologists to PC teams are often for older ICU patients at end of life.^40,41^ Another focus of our curriculum was around encouraging GOC conversations early in the disease course. The presurvey results revealed that the residents did not engage in early GOC conversations. Throughout this curriculum, we stressed the importance of early and ongoing conversations and discussed their association with better outcomes for patients and families.^42-44^ After the training, more residents reported starting GOC conversations earlier in the disease course, which reflects an overall change in behavior.

Our study has several limitations. First, the responses to the postsurvey were limited. Although 50% of the residents completed the postsurvey, the majority did so only 1 month after the session. This greatly limits our ability to determine if their attitudes and behaviors changed longitudinally. We also recognize that the postsurvey respondents may be a select group with positive feelings about this exercise. Second, we conducted the presurvey anonymously which limited our ability to match postsurvey results to individual respondents which hinders our ability to determine the extent of the benefit of the curriculum. Ideally, we would keep all responses anonymous but assign a code to identify individual responses to allow comparisons. Finally, although we were able to show some changes in behavior, we were not able to evaluate interactions with real patients and families following the simulation and were not able to evaluate at the highest Kirkpatrick level.

We were able to recruit faculty and conduct this curriculum for 3 years, which speaks to its feasibility. Although we recognize that it was tailored to the needs of our residency program and focused on patients with ischemic stroke, we do feel that this curriculum is adaptable to other programs and scenarios by modifying the case and with minor adjustments of the introduction and training of the personnel. We also used actors which not all institutions may have access to, but this could be conducted as a role play exercise if needed.

As we resume our curriculum postpandemic, we will train all current residents, and after that, we envision to focus on the end of the PGY 2 year because this timing allows residents some neurology experience and completes the training before they are senior residents and expected to lead GOC discussions. We will also look for ways to introduce further opportunities for PC education and feedback and maybe even propose a mandatory PC experience. Clinical rotations in PC have been shown to foster serious illness communication skills in a wide range of trainees.^45^ We also plan to incorporate a mini-clinical evaluation exercise in which residents use the SICG and receive feedback from trained faculty observers, patients, and family members following GOC conversations. We will recruit and train more faculty facilitators to sustain this program. Finally, we will consider expanding our scope to train neurology attendings who want to improve their skills in leading these complex, emotional conversations.

One of the greatest needs reported during a neuro-palliative care summit in 2017 was teaching and evaluating primary PC skills in residency.^46^ We designed this simulation-based curriculum to address this critical gap in neurology residency education. Our program is feasible, adaptable, and very well received and can serve as a national model for other residencies to prepare the next generation of neurologists to support, care, and advocate for their sickest patients.

## Appendix Authors

**Table:**

Name	Location	Contribution
Marcey Osgood, DO	Department of Neurology, Lahey Health and Medical Center, Burlington, MA	Drafting/revision of the manuscript for content, including medical writing for content; major role in the acquisition of data; study concept or design; analysis or interpretation of data
Brian Silver, MD	Department of Neurology, University of Massachusetts Chan Medical School, Worcester	Drafting/revision of the manuscript for content, including medical writing for content; study concept or design
Jennifer Reidy, MD	Department of Palliative Care, University of Massachusetts Chan Medical School, Worcester	Drafting/revision of the manuscript for content, including medical writing for content; study concept or design
Vandana Nagpal, MD	Department of Palliative Care, University of Massachusetts Chan Medical School, Worcester	Drafting/revision of the manuscript for content, including medical writing for content; major role in the acquisition of data; study concept or design; analysis or interpretation of data

## Glossary

ACGME: Accreditation Council for Graduate Medical Education
GOC: goals of care
IRB: Institutional Review Board
PC: palliative care
PGY: postgraduate year
PPC: primary palliative care
SICG: Serious Illness Conversation Guide

## References

[1] Boersma I, Miyasaki J, Kutner J, Kluger B. Palliative care and neurology: time for a paradigm shift. Neurology. 2014;83(6):561-567. doi:10.1212/WNL.0000000000000674 24991027 PMC4142002

[2] Holloway RG, Arnold RM, Creutzfeldt CJ, et al. Palliative and end-of-life care in stroke: a statement for healthcare professionals from the American Heart Association/American Stroke Association. Stroke. 2014;45(6):1887-1916. doi:10.1161/STR.0000000000000015 24676781

[3] Creutzfeldt CJ, Holloway RG, Curtis JR. Palliative care: a core competency for stroke neurologists. Stroke. 2015;46(9):2714-2719. doi:10.1161/STROKEAHA.115.008224 26243219 PMC4553237

[4] Robinson MT, Barrett KM. Emerging subspecialties in neurology: neuropalliative care. Neurology. 2014;82(21):e180-e182. doi:10.1212/WNL.0000000000000453 24862899 PMC4105252

[5] Carver AC, Vickrey BG, Bernat JL, Keran C, Ringel SP, Foley KM. End-of-life care: a survey of US neurologists' attitudes, behavior, and knowledge. Neurology. 1999;53(2):284-293. doi:10.1212/wnl.53.2.284 10430415

[6] Borasio GD, Weltermann B, Voltz R, Reichmann H, Zierz S. Attitudes towards patient care at the end of life. A survey of directors of neurological departments [in German]. Nervenarzt. 2004;75(12):1187-1193. doi:10.1007/s00115-004-1751-2 15221065

[7] Murray SA, Sheikh A. Palliative care beyond cancer: care for all at the end of life. BMJ. 2008;336(7650):958-959. doi:10.1136/bmj.39535.491238.94 PMC2335219 18397942

[8] Schuh LA, Adair JC, Drogan O, Kissela BM, Morgenlander JC, Corboy JR. Education research: neurology residency training in the new millennium. Neurology. 2009;72(4):e15-e20. doi:10.1212/01.wnl.0000342389.60811.ca 19171823

[9] Accreditation Council for Undergraduate Medical Education. ACGME Program Requirements for Undergraduate Medical Education in Neurology. Revised June 13, 2021.

[10] Mehta AK, Najjar S, May N, Shah B, Blackhall L. A needs assessment of palliative care education among the United States adult neurology residency programs. J Palliat Med. 2018;21(10):1448-1457. doi:10.1089/jpm.2018.0191 30088969

[11] Goyal T, Hasty BN, Bereknyei Merrell S, Gold CA. Education Research: understanding barriers to goals of care communication for neurology trainees. Neurology. 2019;93(8):362-366. doi:10.1212/WNL.0000000000007975 31427487

[12] Goyal T, Robinson MT, Gold CA. Opinion & special articles: competency in serious illness communication for neurology residents. Neurology. 2021;96(12):587-589. doi:10.1212/WNL.0000000000011048 33055275

[13] Creutzfeldt CJ, Gooley T, Walker M. Are neurology residents prepared to deal with dying patients? Arch Neurol. 2009;66(11):1427-1428. doi:10.1001/archneurol.2009.241 19901182

[14] VitalTalk [online]. Accessed June 5, 2023. Vitaltalk.org.

[15] Serious illness care program [online]. Accessed June 5, 2023. ariadnelabs.org/serious-illness-care/.

[16] Center to Advance Palliative Care (CAPC) [online]. Accessed November 15, 2022. capc.org/about/capc/.

[17] Gleicher ST, Hurd CJ, Weisner PA, Mendelson AM, Creutzfeldt CJ, Taylor BL. Curriculum innovations: implementing a neuropalliative care curriculum for neurology residents. Neurol Educ. 2022;1(2):e200021. doi:10.1212/ne9.0000000000200021 PMC12339220 40809501

[18] Goyal T, Bereknyei Merrell S, Weimer-Elder B, Kline M, Rassbach CE, Gold CA. A novel serious illness communication curriculum improves neurology residents' confidence and skills. J Palliat Med. 2023;26(9):1180-1187. doi:10.1089/jpm.2022.0371 36952327

[19] Schuh LA, Biondo A, An A, et al. Neurology resident learning in an end-of-life/palliative care course. J Palliat Med. 2007;10(1):178-181. doi:10.1089/jpm.2006.0089 17298266

[20] Watling CJ, Brown JB. Education research: communication skills for neurology residents: structured teaching and reflective practice. Neurology. 2007;69(22):E20-E26. doi:10.1212/01.wnl.0000280461.96059.44 18040006

[21] Kluger BM, Kramer NM, Katz M, et al. Development and dissemination of a neurology palliative care curriculum: education in palliative and end-of-life care neurology. Neurol Clin Pract. 2022;12(2):176-182. doi:10.1212/CPJ.0000000000001146 35747891 PMC9208408

[22] Carroll E, Nelson A, Kurzweil A, Zabar S, Lewis A. Using objective structured clinical exams (OSCE) to teach neurology residents to disclose prognosis after hypoxic ischemic brain injury. J Stroke Cerebrovasc Dis. 2021;30(7):105846. doi:10.1016/j.jstrokecerebrovasdis.2021.105846 33984743

[23] Kirkpatrick JD, Kirkpatrick WK. Kirkpatrick's Four Levels of Training Evaluation. ATD Press; 2016.

[24] Hospice and Palliative Medicine Competencies Project [online]. Accessed June 5, 2023. aahpm.org/fellowships/competencies.

[25] Nagpal V, Philbin M, Yazdani M, Veerreddy P, Fish D, Reidy J. Effective goals-of-care conversations: from skills training to bedside. MedEdPORTAL. 2021;17:11122. doi:10.15766/mep_2374-8265.11122 33768153 PMC7970639

[26] Hannon P. Neuropalliative care curriculum for neurology residents [online]. Accessed November 17, 2023. aan.com/tools-resources/academic-core-curricula.

[27] Chipman JG, Beilman GJ, Schmitz CC, Seatter SC. Development and pilot testing of an OSCE for difficult conversations in surgical intensive care. J Surg Educ. 2007;64(2):79-87. doi:10.1016/j.jsurg.2006.11.001 17462207

[28] Robinson MT, Holloway RG. Palliative care in neurology. Mayo Clin Proc. 2017;92(10):1592-1601. doi:10.1016/j.mayocp.2017.08.003 28982489

[29] Fallowfield L, Jenkins V, Farewell V, Saul J, Duffy A, Eves R. Efficacy of a Cancer Research UK communication skills training model for oncologists: a randomised controlled trial. Lancet. 2002;359(9307):650-656. doi:10.1016/S0140-6736(02)07810-8 11879860

[30] Moreau D, Goldgran-Toledano D, Alberti C, et al. Junior versus senior physicians for informing families of intensive care unit patients. Am J Respir Crit Care Med. 2004;169(4):512-517. doi:10.1164/rccm.200305-645OC 14656750

[31] Kramer AW, Dusman H, Tan LH, Jansen JJ, Grol RP, van der Vleuten CP. Acquisition of communication skills in postgraduate training for general practice. Med Educ. 2004;38(2):158-167. doi:10.1111/j.1365-2923.2004.01747.x 14871386

[32] Aspegren K. BEME Guide No. 2: teaching and learning communication skills in medicine-a review with quality grading of articles. Med Teach. 1999;21(6):563-570. doi:10.1080/01421599978979 21281175

[33] Kruger J, Dunning D. Unskilled and unaware of it: how difficulties in recognizing one's own incompetence lead to inflated self-assessments. J Pers Soc Psychol. 1999;77(6):1121-1134. doi:10.1037//0022-3514.77.6.1121 10626367

[34] Davis DA, Mazmanian PE, Fordis M, Van Harrison R, Thorpe KE, Perrier L. Accuracy of physician self-assessment compared with observed measures of competence: a systematic review. JAMA. 2006;296(9):1094-1102. doi:10.1001/jama.296.9.1094 16954489

[35] Fox RA, Ingham Clark CL, Scotland AD, Dacre JE. A study of pre-registration house officers' clinical skills. Med Educ. 2000;34(12):1007-1012. doi:10.1046/j.1365-2923.2000.00729.x 11123564

[36] Brewster LP, Risucci DA, Joehl RJ, et al. Comparison of resident self-assessments with trained faculty and standardized patient assessments of clinical and technical skills in a structured educational module. Am J Surg. 2008;195:1-4. doi:10.1016/j.amjsurg.2007.08.048 18082534

[37] Kogan JR, Hatala R, Hauer KE, Holmboe E. Guidelines: the do's, don'ts and don't knows of direct observation of clinical skills in medical education. Perspect Med Educ. 2017;6(5):286-305. doi:10.1007/s40037-017-0376-7 28956293 PMC5630537

[38] Graddy R, Reynolds SS, Wright SM. Coaching residents in the ambulatory setting: faculty direct observation and resident reflection. J Grad Med Educ. 2018;10(4):449-454. doi:10.4300/JGME-17-00788.1 30154978 PMC6108367

[39] Rahmani M. Medical trainees and the Dunning-Kruger effect: when they don't know what they don't know. J Grad Med Educ. 2020;12(5):532-534. doi:10.4300/JGME-D-20-00134.1 33149817 PMC7594774

[40] Kluger BM, Bernat JL. Palliative care and inpatient neurology: where to next? Neurology. 2019;92(17):784-785. doi:10.1212/WNL.0000000000007354 30918099

[41] Taylor BL, O'Riordan DL, Pantilat SZ, Creutzfeldt CJ. Inpatients with neurologic disease referred for palliative care consultation. Neurology. 2019;92(17):e1975-e1981. doi:10.1212/WNL.0000000000007364 30918095 PMC6511082

[42] Mack JW, Cronin A, Taback N, et al. End-of-life care discussions among patients with advanced cancer: a cohort study. Ann Intern Med. 2012;156(3):204-210. doi:10.7326/0003-4819-156-3-201202070-00008 22312140 PMC3616320

[43] Detering KM, Hancock AD, Reade MC, Silvester W. The impact of advance care planning on end of life care in elderly patients: randomised controlled trial. BMJ. 2010;340:c1345. doi:10.1136/bmj.c1345 20332506 PMC2844949

[44] Leung JM, Udris EM, Uman J, Au DH. The effect of end-of-life discussions on perceived quality of care and health status among patients with COPD. Chest. 2012;142(1):128-133. doi:10.1378/chest.11-2222 22241761 PMC3418855

[45] Kurahashi AM, Wales J, Husain A, Mahtani R. Residents' reflections on end-of-life conversations: how a palliative care clinical rotation creates meaningful learning opportunities. Ann Palliat Med. 2020;9(3):738-745. doi:10.21037/apm.2020.03.19 32312060

[46] Creutzfeldt CJ, Kluger B, Kelly AG, et al. Neuropalliative care: priorities to move the field forward. Neurology. 2018;91(5):217-226. doi:10.1212/WNL.0000000000005916 29950434 PMC6093769

